# Cash Transfer to Adolescent Girls and Young Women to Reduce Sexual Risk Behavior (CARE): Protocol for a Cluster Randomized Controlled Trial

**DOI:** 10.2196/14696

**Published:** 2019-12-20

**Authors:** Mwita Wambura, Mary Drake, Evodius Kuringe, Esther Majani, Daniel Nyato, Caterina Casalini, Jacqueline Materu, Deusdedit Mjungu, Soori Nnko, Gaspar Mbita, Esther Kalage, Amani Shao, John Changalucha, Albert Komba

**Affiliations:** 1 National Institute for Medical Research Mwanza Centre Mwanza United Republic of Tanzania; 2 Jhpiego Tanzania - an Affiliate of Johns Hopkins University Sauti project Dar es Salaam United Republic of Tanzania

**Keywords:** adolescent, female, HIV infections/epidemiology, HIV Infections/prevention and control, Herpesvirus 2, humans, incidence, motivation, Tanzania

## Abstract

**Background:**

The HIV epidemic in Eastern and Southern Africa is characterized by a high incidence and prevalence of HIV infection among adolescent girls and young women (AGYW) aged 15-24 years. For instance, in some countries, HIV prevalence in AGYW aged 20-24 years exceeds that in AGYW aged 15-19 years by 2:1. Sauti (meaning voices), a project supported by the United States Agency for International Development, is providing HIV combination prevention interventions to AGYW in the Shinyanga region, Tanzania.

**Objective:**

The aim of this study is to determine the impact of cash transfer on risky sexual behavior among AGYW receiving cash transfer and HIV combination prevention interventions. This paper describes the research methods and general protocol of the study. Risky sexual behavior will be assessed by herpes simplex virus type 2 (HSV-2) incidence, compensated sex (defined as sexual encounters motivated by exchange for money, material support, or other benefits), and intergenerational sex (defined as a sexual partnership between AGYW and a man 10 or more years older). Through a qualitative study, the study seeks to understand how the intervention affects the structural and behavioral drivers of the HIV epidemic.

**Methods:**

The trial employs audio computer-assisted self-interviewing, participatory group discussions (PGDs), and case studies to collect data. A total of 30 matched villages (15 intervention and 15 control clusters) were randomized to either receive cash transfer delivered over 18 months in addition to other HIV interventions (intervention arm) or to receive other HIV interventions without cash transfer (control arm). Study participants are interviewed at baseline and 6, 12, and 18 months to collect data on demographics, factors related to HIV vulnerabilities, family planning, sexual risk behavior, gender-based violence, and HSV-2 and HIV infections. A total of 6 PGDs (3 intervention, 3 control) were conducted at baseline to describe perceptions and preferences of different intervention packages, whereas 20 case studies are used to monitor and unearth the dynamics involved in delivery and uptake of cash transfer.

**Results:**

The study was funded in June 2017; enrollment took place in December 2017. A total of two rounds of the follow-up survey are complete, and one round has yet to be conducted. The results are expected in December 2019 and will be disseminated through conferences and peer-reviewed publications.

**Conclusions:**

This study will document the synergetic impact of cash transfer in the presence of HIV combination prevention interventions on risky sexual behavior among out-of-school AGYW. The results will strengthen the evidence of cash transfer in the reduction of risky sexual behavior and provide feasible HIV prevention strategies for AGYW.

**Trial Registration:**

Clinicaltrials.gov NCT03597243; https://clinicaltrials.gov/ct2/show/NCT03597243.

**International Registered Report Identifier (IRRID):**

DERR1-10.2196/14696

## Introduction

### Background

The HIV epidemic in Eastern and Southern Africa is characterized by a high incidence and prevalence of HIV infection among adolescent girls and young women (AGYW) aged 15-24 years [1,2]. For instance, data from Tanzania, Lesotho, and Mozambique show a prevalence of 1%, 4%, and 7%, respectively, among girls aged 15-19 years, which increased to 3.4%, 24%, and 15%, respectively, among those aged 20-24 years [3,4]. New HIV infections among AGYW in 2015 contributed up to 25% of the new infections among the adult population (>15 years) in East and Southern Africa [5]. AGYW aged 15-24 years in this region have HIV infection rates that are two to four times higher than those of young men of the same age group [1,2,5-7].

Complex interactions between behavioral, biological, and structural factors are part of the explanation for the higher rates of HIV infection among AGYW. Behavioral factors such as intergenerational sex with older male partners who are more likely to be infected with HIV and other sexually transmitted infections (STIs) [8-11], multiple and concurrent sexual relationships [11,12], and transactional sex [11,13] have been shown to increase the risk of HIV acquisition among AGYW. Biological factors related to AGYW have also been shown to be associated with HIV acquisition among AGYW. These include immature cervix [14,15] and increased mucosal HIV exposure time [14,16], which increases the biological susceptibility of adolescent girls. Structural determinants, including poverty and gender inequality, exacerbate vulnerability to HIV infection among AGYW. These determinants affect AGYW’s ability to negotiate condom use, access to STI treatment, and learn skills and access capital required to engage in income-generating activities that will enable them to avoid engaging in transactional sex to meet their daily needs [8,16,17]. Therefore, interventions aimed at addressing AGYW’s HIV vulnerabilities must include a program that addresses structural factors in addition to biomedical and behavioral factors. Such programmatic strategies need to be gender responsive and evidence based to address individual, community, and structural factors contributing to the increased HIV risk in this group [18,19].

There has recently been a call to invest in interventions that help improve the income of vulnerable populations—at individual and household levels [20]. Cash transfer given with or without conditions to recipients has been used to incentivize safe sexual behavior to reduce HIV infection among economically deprived populations [21]. In the context of HIV, cash transfers are provided as a type of structural intervention for HIV prevention, which is generally combined with biomedical and behavioral prevention strategies for HIV infection—an approach commonly referred to as combination prevention [22]. Provision of cash transfer as a strategy for prevention of HIV infections has shown promising results. For example, both conditional cash transfers and unconditional cash transfers were shown to have an impact on some reproductive health outcomes among girls, including prevention of STIs and delaying marriage and childbearing [23,24]. The Zomba study in Malawi demonstrated that cash transfers provided to girls and their parents reduced HIV prevalence by 64% (95% CI 9%-86%) and herpes simplex virus type 2 (HSV-2) prevalence by 76% (95% CI 35%-91%) after 18 months of follow-up. The cash transfers achieved this impact by influencing underlying structural conditions, which, in turn, shaped sexual behavior as well as the risk of HIV infection acquisition [23]. For instance, in the Zomba study, girls in the intervention arm were 79% (95% CI 38%-93%) less likely to have sexual partners aged ≥25 years compared with girls in the control arm [23].

The Sauti (meaning *voices*) project in collaboration with the Government of United Republic of Tanzania and civil society organizations (CSOs) are providing community-based HIV combination prevention interventions among AGYW and other vulnerable populations in selected regions of Tanzania [25]. These regions include Mtwara, Kilimanjaro, Arusha, Shinyanga, Tabora, Singida, Dodoma, and Morogoro. Others are Dar-es-Salaam, Iringa, Njombe, Songwe, and Mbeya. Sauti project is described in detail elsewhere [26]. The CSOs provide reach, spread, and ability to engage the most vulnerable AGYW in the community. Sauti aims to address the biomedical, behavioral, and structural influences on AGYW’s vulnerability to HIV infection by implementing community-based HIV combination prevention interventions among AGYW aged 15-24 years. Sauti’s core package of interventions includes risk reduction counseling; HIV testing services; condom use skills and provision; family planning counseling and service provision; screening and treatment for STIs, gender-based violence, and tuberculosis; and alcohol and drug abuse screening and referral to services. Others are social and behavior change communication (SBCC) training sessions and economic empowerment community banking, also called the Women Organizing Resources Together Plus (WORTH+) intervention and cash transfer program (CTP). The WORTH+ intervention includes financial literacy training, which aims to build microbusiness development skills and community banking. The objectives for SBCC and WORTH+ training are detailed in Table 1.

The CTP under the Sauti project is provided to AGYW through the Determined, Resilient, Empowered, AIDS-free, Mentored and Safe (DREAMS) partnership [27]. The DREAMS partnership is an ambitious endeavor aimed at reducing HIV infections among AGYW in 10 sub-Saharan African countries through a package of health, educational, and social interventions. The partnership aims to address the structural drivers, which increase the risk of HIV infection, including sexual violence, poverty, gender inequality, and lack of education. It aims to transform AGYW in the partner countries into DREAMS women. The Sauti project is one of the primary implementing partners for community-based interventions under the DREAMS initiative in Tanzania. As part of the DREAMS partnership, Sauti is delivering cash transfer for 18 months to 12,144 AGYW who are out of school, aged 15-23 years, and who have completed 10 hours of SBCC training.

CTP villages (or neighborhoods/*mitaa* in urban settings) were identified by the Sauti project, regional and district health authorities, and other stakeholders as communities with high risk of HIV infection and economic and social vulnerability among AGYW who are out of school. The CTP is implemented in four districts of Shinyanga region (Shinyanga Municipal Council, Msalala District Council, Kahama Town Council, and Ushetu District Council) and Kyela district in Mbeya region. Each of the eligible AGYW in the five districts receive 70,000 Tanzanian Shillings (approximately US $33) delivered through mobile money quarterly.

This study describes the research methods and general protocol for a study aiming to evaluate the impact of cash transfer, delivered in the presence of other sexual and reproductive health interventions offered by Sauti project, in the reduction of risky sexual behavior among AGYW. Previous trials in sub-Saharan Africa were conducted to assess the effect of cash transfer [23,28], but none were conducted among out-of-school AGYW to ascertain the synergetic effect of cash transfer, WORTH+, and biomedical and behavioral interventions. The findings will inform scale-up of Sauti interventions among AGYW in Tanzania and potential adoption for HIV programming in sub-Saharan Africa among AGYW.

**Table 1:** Objectives of the social and behavior change communication and Women Organizing Resources Together Plus training sessions.

Objectives for SBCC^a^	Components of WORTH+^b^ training	Objectives for WORTH+
To identify harmful gender norms, especially norms that perpetuate gender inequality and GBV^c^. Identify and explain support systems available for GBV survivors and explain how gender and power are related.	Financial literacy	Explain basic business skills, credit management and bookkeeping, group facilitation and leadership, and conflict resolution; calculate profit, risk, product value, and identify and build a selling advantage, manage capital for growth, and monitor business health in general.
To explain sexually transmitted infections including HIV, HIV prevention methods, and SRH^d^.	SBCC	As explained under SBCC.
To describe family planning methods and identify SRH services available. To communicate with their partners and community to increase awareness for HIV/AIDS and participation in HIV prevention interventions. To understand assertive communication.	Positive parenting	Identify positive parenting and children’s behavior; understand factors that contribute to negative and positive behaviors, types of children’s growth, how to assist groups with special needs, and how to be a role model to the child by showing positive ways of living.

^a^SBCC: social and behavior change communication.

^b^WORTH+: women organizing resources together plus.

^c^GBV: gender-based violence.

^d^SRH: sexual and reproductive health.

### Aims and Objectives

The overall aim of this study is to determine the impact of cash transfer on risky sexual behavior among AGYW receiving biomedical, behavioral, and other structural interventions in Shinyanga region. The primary objective is to assess the impact of cash transfer on the incidence of HSV-2 after 18 months. Secondary objectives are to examine the impact of cash transfers on compensated sex (defined as sexual encounters motivated by exchange for money, material support, or other benefits) and intergenerational sex (defined as a sexual partnership between AGYW and a man 10 or more years older). Through a qualitative study, the study seeks to understand how the intervention impacts the structural and behavioral drivers of the HIV epidemic.

**Figure 1:**
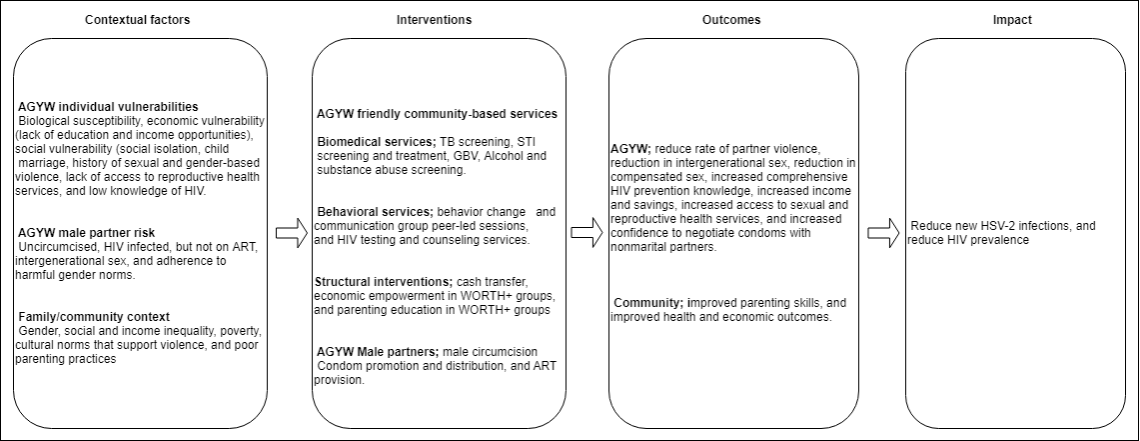
Cash transfer conceptual framework to guide the evaluation work. AGYW: adolescent girls and young women; ART: antiretroviral therapy; GBV: gender-based violence; HSV-2: herpes simplex virus type II; STI: sexually transmitted infection; TB: tuberculosis; WORTH+: women organizing resources together plus.

The theory of change guiding this assessment is presented in Figure 1. It presents the contextual factors contributing to the HIV vulnerability among AGYW, intervention components, outcome, and impact of the interventions. It is hypothesized that cash transfer will reduce risky sexual behavior and new HIV infections among AGYW by boosting their income and therefore increasing their consumption capacity and choices and savings and asset accumulation as well as mitigating the impact of shocks. This will, in turn, reduce risky sex including compensated and intergenerational sex, thereby contributing to a reduction in new STIs.

## Methods

### Study Design and Setting

This study is a two-arm cluster randomized controlled trial (RCT) implemented among AGYW with 1:1 allocation ratio, which employs mixed methods for data collection. The study is conducted in 30 clusters/communities (15 intervention and 15 control) with similar settings across three neighboring districts, namely, Kahama Town Council and Ushetu and Msalala District Councils in Shinyanga region in mainland Tanzania. A cluster or community is defined as an administrative area of the village in a rural setting or neighborhood *(mtaa)* in the urban setting. In this study, clusters rather than individuals were randomized to minimize dilution of the intervention, as the funds transferred were more likely to be shared among family members if they are allocated in different arms [29,30]. The study was retrospectively registered (trial registration: NCT03597243) on ClinicalTrials.gov, because the authors were unaware of the definition of the clinical trial per International Committee of Medical Journal Editors, as this study evaluated interventions provided by Sauti project. All future related trials will be prospectively registered.

### Eligibility Criteria for Clusters

Clusters in the selected councils were eligible for the trial if they were receiving Sauti interventions other than cash transfer and had at least 110 AGYW aged 15-23 years who were out of school according to the household survey conducted in all villages/*mitaa*, which were identified as potential villages to receive the CTP. Eligible clusters were randomized before implementation of the CTP.

### Randomization and Blinding

**Figure 2:**
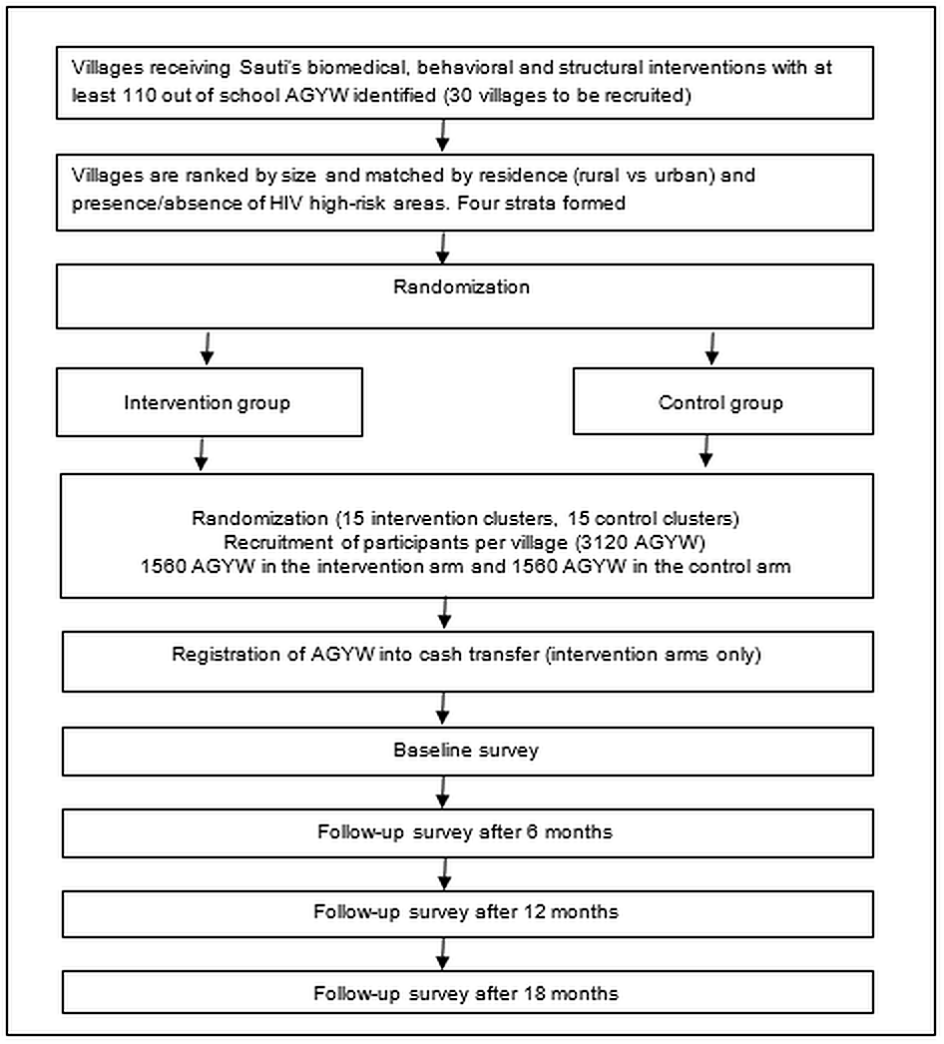
CARE study design. AGYW: adolescent girls and young women.

All clusters that fulfilled the eligibility criteria in the three councils (Kahama town, Msalala district, and Ushetu district) were first ranked by their size and matched by residence (rural vs urban areas) and presence/absence of HIV high-risk areas (mines, plantations, and fishing areas). Therefore, four strata (rural cluster in the high-risk area, urban cluster in the high-risk area, rural cluster in the low-risk area, and urban cluster in the low-risk area) were formed. A total of 15 matched clusters with similar size were selected randomly to participate in the study across the four strata. In each matched pair, one cluster was randomly selected to receive the intervention package, and the other was automatically assigned to receive the control package. Matching was done to minimize the between-community variance in HSV-2 incidence within the matched clusters. No blinding was performed in this study, as it was not possible to blind study participants, Sauti implementers, data collectors, and data analysts because the intervention provided (cash transfer) was known to study participants, implementers, and researchers. Figure 2 presents an overview of the study design.

### Participant Recruitment

In villages/*mitaa* identified as potential recipients of the cash transfer, the Sauti project conducted a household survey to identify out-of-school AGYW. Only AGYW identified through this survey constituted the population eligible for CTP and the study. This way, only AGYW who were already out of school before the initiation of the project were enrolled in the study. Following this survey, community meetings of AGYW and their caregivers were held as part of the Sauti project to introduce the study. Eligible AGYW were invited to attend SBCC training sessions. Eligible participants were as follows: AGYW aged 15-23 years, graduated from 10 hours of Sauti’s SBCC sessions, and resident of the village of recruitment. Other inclusion criteria were as follows: registered into CTP (applicable to those in the CTP areas only) and currently out of school (not enrolled in primary, secondary, or tertiary education; they either have never been to school or have dropped out of school at least 1 month before study enrollment), as documented through a previous household survey of the CTP. In addition, AGYW who were willing and able to give voluntary, informed consent/assent to all study procedures, including HIV and HSV-2 testing and receiving test results, were considered eligible. Consent from parent /guardian was required for AGYW who are younger than 18 years, with the exception of emancipated minors (ie, minors who are married, have given birth, or demonstrating full independence, eg, living alone or heading the household). AGYW found to be HIV- and HSV-2 positive at baseline or follow-up visit were not excluded from the study because excluding them is likely to disclose their HIV or HSV-2 serostatus to the community and therefore expose them to stigma and other social harms. Their data will also contribute to the analysis of the study behavioral outcomes (reporting of compensated sex and intergenerational sexual partnerships).

During SBCC sessions in the study clusters, AGYW were given information about the study. After the 10 hours of SBCC sessions, AGYW were informed about the study enrollment through information provided in their groups. This way, the potential participants were aware of dates of recruitment and location of the study site. During recruitment into the RCT, potential participants approached the registration desk for prescreening consent and eligibility screening. This process continued until the desired sample was reached.

Participatory group discussions (PGDs) and case study participants were selected purposively among the AGYW enrolled in the RCT using the baseline audio computer-assisted self-interviewing (ACASI) data. For PGD participation, participants were selected purposively to ensure maximum variability of their characteristics. Such characteristics included young versus old, never been to school versus completed primary school, heads of household versus nonheads of households, and teen pregnancy versus never been pregnant. Each PGD had 8-12 participants. For case studies, participants were selected purposively, following the experience of at least two of the following characteristics of interest: intimate partner violence, teenage pregnancy (pregnancy before 18 years of age), and living alone or as head of household.

### Intervention Description

AGYW in the intervention arm received unconditional cash transfer in quarterly installments of 70,000 Tanzania Shillings (approximately US $33) through mobile money on their cellular phones plus biomedical, behavioral, and other structural interventions. The control arm did not receive cash transfer but received the other Sauti project interventions.

The first installment of cash transfer was released after registration into the CTP and completion of the baseline survey. The cash transfer was delivered for 18 months, whereas other Sauti interventions were delivered for the entire duration of the project (5 years). Table 2 presents the biomedical, behavioral, and structural interventions as defined by the Joint United Nations Programme on HIV/AIDS [31].

**Table 2:** Proposed components of the interventions in the control and intervention arms.

Strategy	Control arm	Intervention arm
Biomedical interventions	Biomedical community-based health services include HTS^a^, condom skills and provision, tuberculosis screening, STI^b^ screening, GBV^c^ screening, alcohol and substance abuse screening. Escorted referrals are provided to GBV survivors and the GBV desk for social, legal, and medical services. Escorted referrals are also provided to HIV-positive AGYW^d^ to care and treatment clinics. Other services include FP^e^ counseling and service provision and screening for nutritional assessment and counseling support.	Same as the control arm
Behavioral interventions	SBCC^f^ group peer-led sessions designed to address the significant determinants of HIV risk, gender, and reproductive health. Community-based HIV testing and counseling services, which include risk reduction counseling provided alongside HTS. SBCC sessions include gender, gender and power issues, understanding sexuality, sexual and reproductive health systems, effective communication, STIs and HIV, risky behavior, contraception, GBV, and respectful relationships. The minimum package is 10 hours of sessions. In selected communities, AGYW receive posttest services and Alcoholics Anonymous programs.	Same as the control arm

Structural interventions	AGYW participates in WORTH+^g^ economic empowerment, health (HIV and FP) education, and parenting education groups. The WORTH+ package sessions are as follows: financial literacy sessions, dream and develop self-goals, literacy skills exercise, savings, safe money handling, loans, proper use of loans, dividends and village bank cycle, successfully selling, managing capital, and building business. Other packages in the WORTH+ package include parenting sessions, understand children, and learn parenting and being a good example to children. WORTH+ groups meet weekly and include social cohesion activities. In addition, the SASA! GBV program works with communities to address adverse gender social norms.	Same as the control arm plus cash transfer

^a^HTS: HIV testing services.

^b^STI: sexually transmitted infection.

^c^GBV: gender-based violence.

^d^AGYW: adolescent girls and young women.

^e^FP: family planning.

^f^SBSS: social and behavior change communication.

^g^WORTH+: women organizing resources together plus.

### Data Collection

The study uses both quantitative and qualitative methods to evaluate the impact of the intervention on primary and secondary outcomes. The impact evaluation study collects data through the following components.

#### Sauti Routine Program Monitoring Data

Sauti routine program monitoring data will be used to evaluate the uptake of biomedical, behavioral, and structural interventions including the CTP. The routine program monitoring data are collected continuously throughout the program period and obtained from the program only for research participants who provided consent for their data to be used for research purposes. The data collected from the program will be used to describe the context under which cash transfer and Sauti interventions are implemented and understand the reach and coverage of interventions provided by Sauti. Specifically, the routinely collected data will be used to show fidelity (the extent to which the intervention is delivered as intended) [31] and acceptability (rate of intervention uptake, adherence to the package provided, retention, and attrition).

#### Cluster Randomized Trial

The baseline assessment was conducted following enrollment into the cash transfer program in a confidential environment in preidentified venues in the respective villages. Following the informed consent procedures and eligibility assessment, a structured self-administered questionnaire was administered to trial participants using ACASI. Data were collected on demographics, factors related to HIV vulnerabilities, family planning, sexual risk behavior, and GBV. All trial participants were asked to give a blood specimen for HIV and HSV-2 infection determination. Participants underwent pretest counseling, HIV test, and receive HIV test results on the same day after posttest counseling according to national HIV testing algorithms. HSV-2 infection determination was conducted at the National Institute for Medical Research (NIMR) Mwanza laboratory using the enzyme-linked immunosorbent assay technique, and the results were communicated to the participants within 2 weeks. HSV-2 test results include posttest counseling and referral for treatment of HSV-2, where needed.

Following the delivery of the intervention in the intervention clusters, the first follow-up survey (6 months after the baseline), second follow-up (12 months after the baseline), and third follow-up (18 months after the baseline) are undertaken in both clusters over 1 month. Follow-up surveys are performed in a preidentified venue used during the baseline assessment. Participants’ data and blood specimen are collected at follow-up visits (6, 12, and 18 months) as well. Data and blood specimens collected are linked to the AGYW by a unique participant identification number. The interview is conducted in a private area to ensure confidentiality and anonymity of the information collected.

#### Qualitative Components

PGDs and case studies are used to collect qualitative data. PGDs describe perceptions and preferences of different intervention packages, whereas case studies are used to track in detail a small cohort of 20 AGYW purposely selected to monitor and unearth the dynamics involved in delivery and uptake of cash transfer. PGDs are used to collect data during the baseline survey, whereas case studies follow-ups selected participants in the intervention arm only at more regular intervals (every 6 months).

A total of 6 PGDs (3 intervention and 3 control PGDs) are conducted at baseline. Each PGD comprises 8 to 10 AGYW. Participants for the PGDs are randomly selected and approached to participate. Inclusion criteria for the PGDs include willingness to participate in a PGD; consent from the participant and parent or guardian, if applicable, as documented by written informed consent; and enrollment in the trial with complete baseline ACASI questionnaire.

Qualitative data gathering and analysis are performed as a continuous, flexible, and iterative process. Preliminary data collected through case studies are analyzed in the field (sequential analysis), after which further analysis of texts is performed to confirm or refute interim results through constant validity checks until saturation is reached.

### Outcomes

End points are assessed at baseline and after every 6 months over the next 18 months. The primary outcome measure is the incident HSV-2 infection. Secondary outcome measures include HIV prevalence, reporting of intergenerational sex (sexual partnership between AGYW and a man 10 or more years older), and reporting of compensated sex (AGYW are asked to report whether they have received money or gifts in exchange for sex) in the last 6 months. Compensated sex is assessed by self-report of whether AGYW has had sex with anyone because they received money or gifts or because they expected to receive money or gifts. The primary exposure is cash transfer received. Table 3 presents the outcomes, indicators of change of outcomes, and uptake of interventions.

**Table 3:** Outcomes, indicators of change of outcomes, and uptake of interventions.

Factor	Variables
Primary and secondary outcomes	Herpes simplex virus type 2 incidence, HIV prevalence, reporting of intergenerational sex, Compensated sex
Indicators for change	Increased comprehensive HIV prevention knowledge, increased income and savings, increased confidence to accept an HIV test, increased confidence to negotiate condoms with nonmarital partners, and increased access to biomedical interventions (community-based HIV testing and counseling services plus, sexually transmitted infection, gender-based violence, and substance and alcohol screening and family planning)
Uptake of Sauti interventions	Individuals who received interventions or control packages; within the intervention sites, individuals who received (1) SBCC^a^, cash transfer, WORTH+^b^ and (2) cash transfer and combination prevention interventions; and within the control sites, individuals who received (1) SBCC and WORTH+ and (2) combination prevention interventions

^a^SBCC: social and behavior change communication.

^b^WORTH+: Women Organizing Resources Together Plus.

### Data Management

The data from ACASI and qualitative interviews are collected using tablets and digital audio recorders, respectively. Data are directly transferred to the NIMR cloud storage at the end of each working day. The site supervisor synchronizes the data to the cloud. A specialized data person with permission to access data in the cloud extracts all study data and uploads them to NIMR data management system for further data cleaning and production of analytical datasets. All sound files are deidentified, transcribed, and translated before analysis.

All quantitative data are managed and cleaned in Stata (StataCorp, College Station, Texas) version 13 [32], whereas qualitative data analyses are cojointly performed by coinvestigators using NVivo software (QSR International, Melbourne, Australia) [33]. Data validation checks to identify errors and inconsistencies are programmed and run in Stata. Data queries are raised on data clarification forms and sent to the field to the appropriate member of the study team to be resolved; when a query is resolved, the database is updated accordingly. After data are clean, ACASI and laboratory data are merged using the unique study number. All electronic data are stored in password-protected database systems. There is also hardware password protection on computers, servers, and networks. All printed transcripts and paper-based forms are securely locked up in the filing cabinet with limited access.

### Statistical Analysis

#### Sample Size

CRT is powered to detect intervention impact on the reduction of new HSV-2 infections. HSV-2 incidence was chosen as a proxy measure for HIV incidence, as the study would not be powered to detect a difference in HIV incidence in a relatively low-incidence setting such as Tanzania. A study conducted among women working in the food and recreational facilities, in a setting similar to one in this study, found an HSV-2 incidence of 35.2 per 100 person-years and 24.2 per 100 person-years among women aged 18-19 years and 20-24 years, respectively [34].

All sample size calculations were performed using methods for matched cluster randomized trials. A sample of 15 paired clusters (30 clusters) with 70 AGYW per cluster achieves >80% power to detect a difference of –0.07 between the intervention HSV-2 incidence of 13% and the control HSV-2 incidence of 20% in an 18-month period. The between-community coefficient of variation in HSV-2 incidence was assumed to be 0.25 [35], and the significance level of the test is 0.050. The sample size (70 AGYW per cluster) has been increased by 48% to 104 AGYW per cluster (1560 per arm) to account for attrition rate and background HSV-2 prevalence. It is estimated that, at baseline, HSV-2 prevalence will be 20% among AGYW aged 15-23 years [34]; the attrition rate is 10% and the nonresponse rate is 18% over 18 months.

### Statistical Methods

The primary analysis will be the difference in outcome measure (proportion of HSV–2-negative AGYW who are still free of HSV-2 after 18 months of follow-up) from each matched pair of clusters across the two arms. Secondary analysis will report cumulative incidence rates using survival analysis techniques. We hypothesize that the communities receiving cash transfer will have a significantly lower cumulative incidence of HSV-2 than that of the villages not receiving the cash transfer.

HIV prevalence will be assessed by comparing the prevalence in the intervention arm against the prevalence in the control arm. The primary analysis will be the proportion of AGYW who are HIV+ at 18 months across the two arms. Secondary analysis will be performed using logistic regression analysis, adjusting for clustering and baseline HIV measurements. Cash transfer recipients are expected to have a significantly lower HIV prevalence than those in the control arm. A comparison of sexual behavior outcomes between the two arms will be made by a repeated measure logistic regression. The logistic model adjusted for clustering will be used to account for the clustering design.

Qualitative data will be analyzed following the grounded theory approach [36]. Themes will be identified through coding. Open codes will be used to capture a priori and emerging concepts. Open codes will be developed by two researchers after independently reviewing a limited number of transcripts based on emerging concepts. Following initial code development, the two researchers will compare themes and codes and develop a final coding scheme. The research team will meet to discuss emerging themes. After completing the coding for half of the transcripts, axial coding (ie, comparing code categories to the objectives of the study and findings from literature) will be applied to group open codes into abstract conceptual categories. In all, 10% of the transcripts will be double coded in NVivo to ensure consistency between coders. The two coders will discuss and agree upon quotations from participants that best represent the themes. The coding scheme will be documented in a codebook.

Queries will be performed in NVivo to analyze themes across participants to understand (1) how the desired impact was achieved by the interventions implemented and (2) the perceptions and acceptability of the interventions.

### Secondary Analysis

This study is collecting extensive data on demographics; the prevalence of HSV-2 and HIV infections; uptake of biomedical, behavioral, and structural interventions; and risk factors for HIV and HSV-2 infections among AGYW who are out of school. Secondary analysis is planned to understand how structural factors influencing vulnerability to HIV infection influence the uptake of cash transfer and other interventions and to understand how cash transfer may impact risky sexual behavior. Analysis of the longitudinal data will also be performed to explore how cash transfer impacts indicators for change and ultimately changes the outcome measure over time. The generalized estimating equations will be used for causal analysis and to verify the abovementioned theory of change.

### Retention

To ensure that research participants recruited into the study are retained, the study developed strategies to optimize retention and minimize attrition rate. First, all participants in the intervention and control communities were given phones for maintaining regular contact with researchers. Second, a detailed and thorough explanation of the study visit timeline (baseline and all follow-up rounds) was given during the informed consent process. Third, during enrollment, participants were requested to provide phone numbers of their friends or relatives to facilitate tracing in the follow-up surveys. The phone numbers were updated in the follow-up rounds. Short text messages were sent before and during the time of data collection to remind and emphasize the importance of study participation in the success of the study. Fourth, the research team is working closely with CSOs, which train and support AGYW. AGYW receiving Sauti interventions in the village are trained and supported by 1-3 empowerment workers who are employees of the CSOs.

### Safety Monitoring

Given that this is an impact-evaluation study conducted among participants receiving Sauti’s standard biomedical, behavioral, and structural interventions, an interim analysis is not planned. For the same reason, a data and safety monitoring board is not established. This idea was approved by the institutional review boards, which granted ethics approval to the study. It is anticipated that any harm to AGYW as a result of study participation will be minimal. The study adheres to all safety guidelines established by Sauti program in defining and reporting social harm events. The social harm events resulting from study participation are closely monitored; recorded in the social harm event form; and reported to the Sauti program, CSOs, and the study principal investigator who initiates further investigations and responses, as appropriate.

### Auditing

Members of the study team, representatives of the CSOs implementing the study, and the sponsor conduct regular auditing of the study activities. During auditing, a review of the informed consent forms and study-related materials and procedures is performed to ensure that informed consent forms are thoroughly and accurately filled and that all the consenting procedures are strictly adhered to. A review of study-related materials and procedures is performed to ensure that the study is conducted and reported in compliance with the protocol, good clinical practices, and ethics requirements.

### Ethics

This study was approved by the Medical Research Coordinating Committee of NIMR in Tanzania (NIMR/HQ/R.8c/Vol.II/841) and institutional review board of the Johns Hopkins University (IRB00007976). Approval to conduct the study in the study communities was also provided by regional and district authorities as well as community leaders.

All AGYW graduating from SBCC sessions in the study clusters are informed about the study in group introductory session, and study information was provided in oral and written forms using Swahili language. Girls who required consent from parents/guardian were advised to bring their parents/guardians, if interested in study participation. All girls younger than 18 years of age required consent from parents/guardian and their assent to participate in the study, except for emancipated minors (ie, minors who are married, have given birth or demonstrating full independence, eg, living alone or heading the household). If a mature participant or guardian is illiterate, the consent procedure is conducted in the presence of a witness who is literate and not related to the study. AGYW willing to participate received further information on a one-to-one basis in a confidential environment guided by the participant information and informed consent form. As part of the consent process, study staff explained the study aims, procedures, any inherent risks or benefits, and their right to decline study participation or terminate participation in the study at any time, without giving reasons and affecting their health care or participation in the CT program provided by Sauti. Participants and their parents/guardians were given time to discuss participation and raise any additional questions. Consent to study participation was documented using a written consent form before any study-related data collection. Illiterate participants and parents/guardians provided a thumbprint in the presence of a witness who signed the consent.

### Confidentiality

AGYW’s names, their telephone numbers, and any locator information collected that identifies them are kept confidential and always under lock and key whenever not in use. Only study personnel involved in data collection have access to locator information. Locator information is only used to ensure validation of the identity of the participant in follow-up visits and for contacting the participant for participation in the follow-up rounds.

A unique study identification number and the encrypted Sauti routine identification (SRI) number (participant unique identifier of the Sauti program) are collected using the study tools and hence captured in the database. The SRI number is encrypted before it is used for study purposes of maintaining the confidentiality of participant’s information. Although the study number is used to identify the dataset for an individual participant in the study dataset, the SRI is used to link study data collected and routine data obtained from the program.

### Participants and Public Involvement

This is an impact evaluation study of the interventions provided by Sauti project. AGYW were involved in the design of this study through their representative CSOs. The involvement included advising on the content of the study materials, data collection techniques such as the use of PGDs, and techniques that enhance confidentiality and anonymity (eg, ACASI). CSOs were also crucial in the recruitment of study participants and are involved in monitoring study activities and tracing of study participants. They will also be involved during the dissemination of the findings at the community and regional level.

During the development of interventions, the Sauti project involved intensive dialogues with local leaders, Ministry of Health, Community Development, Gender, Elderly and Children, Tanzania Social Action Fund, National AIDS Control Program, Tanzania Commission for HIV/AIDS, Tanzania Communications Regulatory Authority, mobile communication companies, CSOs, and AGYW representatives among other national and international stakeholders. These dialogues led to the development of the combination prevention interventions offered by the project to AGYW, including the cash transfer amount and payment modality.

## Results

The study was funded in June 2017, and the enrollment took place in December 2017. The baseline study was conducted among AGYW who consented for study participation, and study participants were followed up for 18 months. Follow-up study assessments are conducted 6, 12, and 18 months postintervention initiation. A total of two rounds of the follow-up surveys postintervention are complete, and one round has not yet been performed. The results are expected in December 2019 and will be disseminated through conferences and peer-reviewed publications.

## Discussion

### Overview

This cluster randomized trial intends to evaluate the impact of cash transfer in the presence of combination prevention interventions in the reduction of risky sexual behavior. Previous trials in sub-Saharan Africa were conducted to assess the effect of cash transfer [23,28], but none were conducted among out-of-school girls to ascertain the synergetic impact of cash transfer, WORTH+ interventions, and biomedical and behavioral interventions.

### Study Strengths and Limitations

This study has several strengths. First, the study incorporates both qualitative and quantitative data collection techniques to understand how cash transfer and other interventions synergistically mediate to reduce risky sexual behavior. Second, the study uses ACASI to collect sexual behavior and other sensitive data. Studies comparing face-to-face interviews with ACASI have reported that respondents are more likely to be open and honest when using ACASI [37,38]. Third, the study collects longitudinal data on biomedical, behavioral, and structural interventions on a large sample of AGYW (n=3120) who are out of school and therefore vulnerable to HIV infection to allow for the detection of the difference between arms. However, the study clusters are selected from Shinyanga region, which has a prevalence of HIV above the national average and may not be generalizable to other study regions with lower HIV prevalence in Tanzania.

### Conclusions

This study aims to document the synergetic impact of cash transfer in the presence of other biomedical, behavioral, and structural interventions on risky sexual behavior among out-of-school AGYW. The results will strengthen the evidence of cash transfer in the reduction of risky sexual behavior and provide feasible HIV-prevention strategies for vulnerable AGYW.

## Appendix

Multimedia Appendix 1 Consort-EHEALTH checklist (V 1.6.1).

## Abbreviations

ACASI: audio computer-assisted self-interviewing
AGYW: adolescent girls and young women
CSO: civil society organization
CTP: cash transfer program
DREAMS: Determined, Resilient, Empowered, AIDS-free, Mentored and Safe’ women
GBV: gender-based violence
HSV-2: herpes simplex virus type 2
NIMR: National Institute for Medical Research
PGD: participatory group discussion
RCT: randomized controlled trial
SBCC: social and behavior change communication
SRI: Sauti routine identification
STI: sexually transmitted infection
WORTH+: women organizing resources together plus
